# Gender-specific responses in gene expression of Nile tilapia (*Oreochromis niloticus)* to heavy metal pollution in different aquatic habitats

**DOI:** 10.1038/s41598-024-64300-4

**Published:** 2024-06-25

**Authors:** Simone T. Awad, Shabaan A. Hemeda, Abeer F. El Nahas, Eman M. Abbas, Mohamed A. S. Abdel-Razek, Mohamed Ismail, Ahmed Mamoon, Fawzia S. Ali

**Affiliations:** 1 National Institute of Oceanography and Fisheries, NIOF, Cairo, 11516 Egypt; 2 Department of Animal Husbandry and Animal Wealth Development, Faculty of Veterinary Medicine, Alexandria University, Abees 10th District, P.O.: 21944, Alexandria, Egypt; 3 Department (Chemistry and Toxicity) of Pesticides, Faculty of Agriculture, Kafrelsheikh University, Kafr El-Sheikh, Egypt; 4 Genetics Department, Faculty of Agriculture, Menoufia University, Shibin El‑Kom, Egypt; 5 Fish Production Department, Faculty of Agriculture-Al-Azhar University, Nasr City, Cairo, 11884 Egypt

**Keywords:** Gene expression, Heavy metal pollution, Lake Nasser, *Oreochromis niloticus*, Gender-specific expression, Ecology, Genetics, Immunology

## Abstract

Monitoring heavy metal accumulation is essential for assessing the viability of aquatic ecosystems. Our methodology involved integrating analysis of immunological, stress, inflammatory, and growth-related gene expression in male and female Nile tilapia with on-site recordings of physicochemical parameters. Additionally, we assessed the effect of different physicochemical parameters on heavy metal bioavailability and residual concentration in fish and water. Samples of fish and water were gathered from three different localities: Lake Brullus, a brackish lake sited in northern Egypt; Lake Nasser, an artificial freshwater reservoir located in southern Egypt; and El-Qanater El-Khayria, a middle-freshwater location belonging to the Rashid branch of the river Nile. The assessment of heavy metal residues (Fe, Cu, Zn, Mn, and Ni) revealed that their concentrations were higher in fish specimens compared to their counterparts in water (except for Ni). In addition, Lake Brullus emerges as the most polluted area, exhibiting elevated levels of heavy metals concentrations in water and fish specimens. In contrast, Lake Nasser showed the least degree of heavy metals pollution. Gene expression analysis revealed gender-specific responses to heavy metal exposure at the three investigated water bodies. The expression of hepatic antioxidant genes (*GST* and *MT*) and inflammatory-related genes (*CC-chemokine* and *TNFα)* increased in males compared to females. In females, the immune and pro-inflammatory-related genes (*IgM* and *CXC2-chemokine*) transcripts were upregulated. Additionally, growth-related genes were downregulated in both Lake Brullus and El-Qanater; on the contrary, fish samples from Lake Nasser exhibited a normal expression pattern of growth-related genes. Stress-related genes (*HSP70* and *HSP27*) showed significant downregulation in gills of both genders from Lake Brullus. The minimal presence of heavy metal contaminants in Lake Nasser seems to endorse the normal patterns of gene expression across all gene categories. A potential gender-specific gene expression response towards pollution was noticed in genes associated with inflammation and antioxidant activities. This highlights the importance of considering gender-related responses in future environmental assessments.

## Introduction

The water ecosystem is a complex and diverse system containing various living and non-living components, including organic and inorganic substances in both soluble and non-soluble forms^1^. The quality of the aquatic environment is established by the interplay between its physical and chemical constituents, such as salinity, temperature, dissolved oxygen, hydrogen ion potential (pH), total dissolved and suspended solids, nutrients, and various pollutants^2^. These factors are the main restrictive impediments for growth and thriving of aquatic biota^3,4^.

Pollution causes an increasingly rapid decline in the quality of freshwater resources^5^. Heavy metals including copper (Cu), manganese (Mn), zinc (Zn), iron (Fe), lead (bp), cadmium (Cd), and mercury (Hg) are a natural component of the earth’s crust, which exist in the aquatic environments through geochemical and natural origins, including rock weathering, erosion, and hydrodynamic processes^6,7^. However, their augmentation due to anthropogenic activities has turned it into one of the main growing concerns worldwide because of their wide variety of compounds, pathways, high toxicity, and long-term stability^6,8,9^. Certain heavy metals, for instance, Cu, Mn, Zn, and Fe are indispensable for biological processes in low concentrations. However, in high concentrations, they can be toxic to both aquatic life and humans. Other heavy metals, for example, lead (bp), cadmium (Cd), and mercury (Hg), are unnecessary for biological processes and are highly poisonous to all creatures, even in minimal concentrations^10^. Once heavy metals enter the ecosystem, they accumulate in sediments and biota and then travel up the food chain, resulting in high concentrations in fish (biomagnification), to which fish respond with great sensitivity^11^. Chronic heavy metals exposure in fish triggers a range of harmful consequences, including morphological, behavioral, haematological, and histological changes. Additionally, they induce immune responses, infertility, lower life expectancy, and developmental retardation^12,13^. Meanwhile, acute exposure to heavy metals significantly affects fish’s nucleic acids, causing DNA damage and mutations^12,14,15^. The eco-environmental risks associated with heavy metals pollution in aquatic ecosystems rely not just on their overall concentrations but also on their bioavailability, which is controlled by the chemical properties of the heavy metals and the surrounding environment^8^.

Egypt harbors a diverse range of aquatic environments, from healthy and nonpolluted to polluted^16,17^. These variable environments can be regarded as reference models for investigating the influence of different concentrations of pollutants (heavy metals accumulation, in particular) on ecosystems’ biota^3,18^. For instance, Lake Nasser, south Egypt, is renowned for its stable water quality and the high growth performance of its fish populations, furthermore, it represents a reference for pre-industrial ecology of the river Nile^18–20^. Therefore, it can provide information about the individual impact of water physicochemical parameters on heavy metal bioavailability. On the other hand, Lake Brullus, a brackish water body that offers insights into the influence of salinity and anthropogenic activities on heavy metal bioavailability, particularly with the significant decline in its fish production over time^21^.

Researchers are now more interested in revealing aquatic heavy metal accumulation by evaluating the expression of some affected genes as biomarkers for pollution while considering sex-dependent variations. This is because there are many factors that interfere with heavy metal bioaccumulation, metabolism, and bioavailability. Additionally, there is a strong interaction between aquatic organisms and their environment, and there are differences in stress and immune responses between fish based on sex. These biomarkers provide early indicators of biological effects instead of chemical biomonitoring of contaminated areas^11,12,22,23^.

In the same context, the current investigation has considered three ecologically diverse water bodies to investigate the intricate interaction between water physicochemical properties and heavy metal bioavailability. The euryhaline Nile tilapia, *Oreochromis niloticus,* was best suited for this study; hence, it is one of the most ubiquitous fish species in the Egyptian ecosystem. Additionally, it is characterized by a great adaptability to water contamination and temperature fluctuations^24,25^.

The current study aimed to address three main objectives: first, to evaluate the water quality in three geographically and ecologically diverse ecosystems in Egypt (Lake Brullus, Lake Nasser, and El-Qanater El-Khayria (River Nile). Second, to investigate the effect of different heavy metal concentrations (Fe, Cu, Ni, Zn, and Mn) on the expression patterns of genes linked to growth, stress, and immunity in fish under diverse water conditions. Finally, to assess if males and females respond differently to varying concentrations of heavy metals, that might indicate a gender-specific impact.

## Materials and methods

### Site description

**Figure 1:**
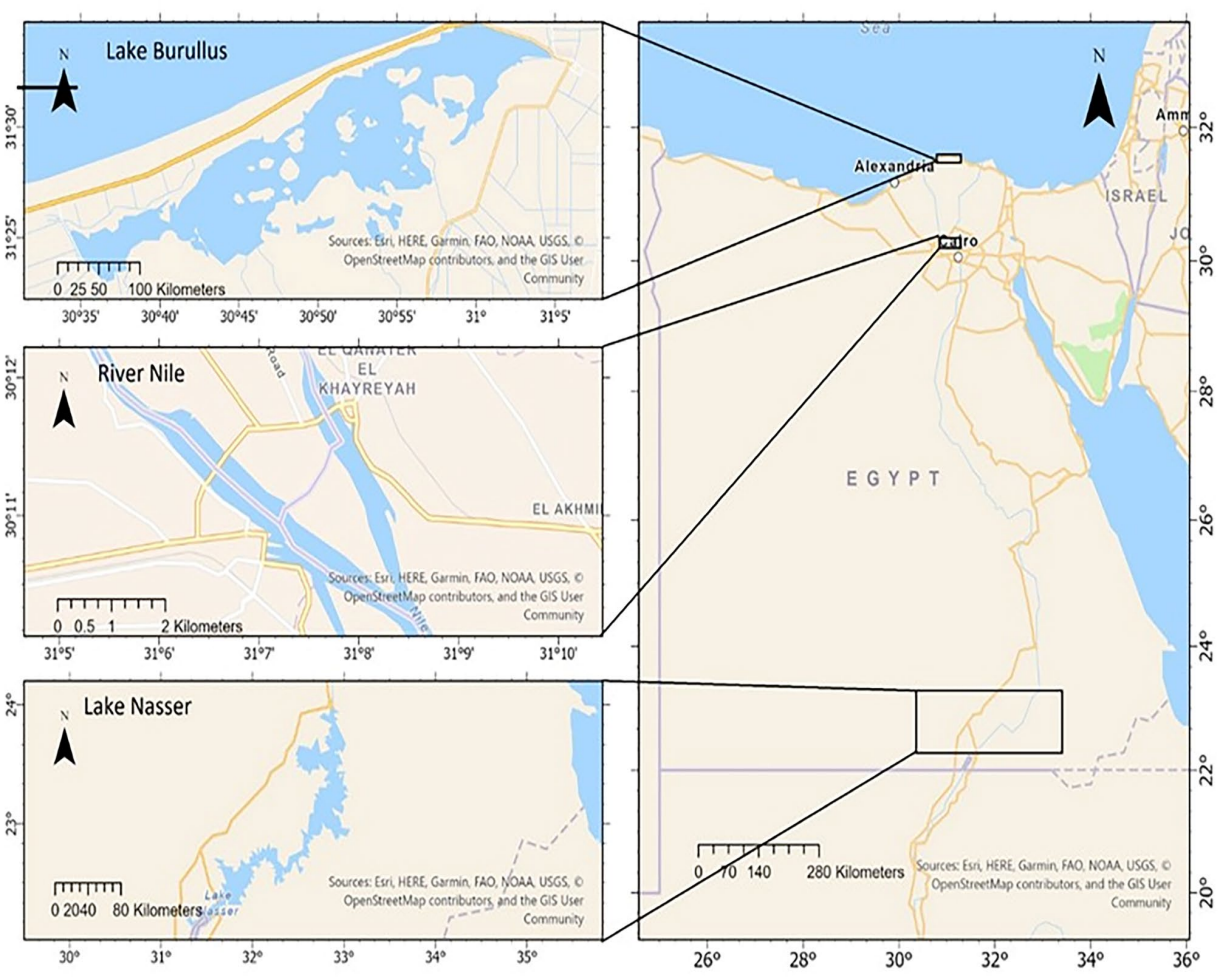
The three studied locations; Lake Brullus, El Qanater El Khayria, and Lake Nasser. The maps were created using ArcGIS Pro 3.1® software by Esri. ArcGIS® and ArcMapTM are the intellectual property of Esri and are used under license. Copyright © Esri. All rights reserved. Esri® software (www.esri.com).

The first site is Lake Brullus, a northern Egyptian brackish water lake (31° 24′ 50′′ to 31° 27′ 50′′ N and 30° 95′ 50′′ to 30° 97′ 50′′ E, Fig. 1) with a confined outlet to the Mediterranean Sea and 370 km^2^ of open water. The lake's eastern portion is the shallowest and most saline, where it is connected to the Mediterranean Sea via a long channel (El-Boughaz Canal). The lake receives vast volumes of drainage and a relatively small volume of seawater near its southern coast. It also receives irrigation water from nine drainages and one freshwater channel^26,27^.

The second location is Lake Nasser, a 5,250-square-kilometer freshwater artificial lake reservoir located in southern Egypt, spanning the border between Egypt and Sudan (21° 00′ N to 23° 00′ N and 30° 00′ E to 33° 00′ E)^28,29^. It represents a semi-isolated ecosystem formed in 1969 owing to the high dam construction^30^. The lake possesses an enormous water storage capacity of 151–165 km3. Lake Nasser is renowned for its stable water quality and the high growth performance of its fish populations^18^. The lake's eastern boundary is the Eastern Desert, and the western border is the Western Desert, which holds several agricultural fields^31^.

The third location is El-Qanater El-Khayria; it is situated at the apex of the Nile Delta, where the river Nile splits into two main streams, the Damietta and Rosetta branches^32^, and it is the location of the Delta Barrage. The Rosetta Branch extends approximately 220 km. It extends from the Delta Barrage to the Edfina Barrage, then discharges the surplus water into the Mediterranean Sea. It receives polluted water from agricultural, domestic, and factory drainage^33^. While the Damietta branch extends about 245 km from the Delta barrage (at 26.5 km behind El-Roda Gouge station) to the Mediterranean Sea, this branch also receives contaminated drainage waters from different sources, including fertilizer plants, electric power stations, food processing factories, and domestic wastes^32^.

### Sample collection

Representative water and fish specimens were gathered from the three locations (Lake Brullus, Lake Nasser, and El-Qanater El-Khayria (River Nile)) by boat equipped with fishing gear (net), guided by GPS, to assess the residual concentration of five heavy metals (Mn, Cu, Fe, Ni, and Zn). Representative samples of live fish (six per location) were also collected for gene expression analysis. The gathered fish from all locations were of similar size with an average weight and length of 145 g and 21 cm. These fish groups were compared to the control group of the same species and similar body measurements, reared at the National Institute of Oceanography and Fisheries (NIOF), El Max Station, in dechlorinated tap water with minimal heavy metals and fed on natural food. All the experimental protocols followed the general guidelines for the handling and usage of laboratory animals, as indicated by the National Institutes of Health (NIH). The study earned approval from the local ethical committee of animal use at the National Institute of Oceanography and Fisheries, Egypt (NIOF-AQ6-F-23-R-045).

### Physicochemical properties of water specimens

During the summer (July 2022), water temperature, salinity, and hydrogen ion potential were measured and recorded at the collection sites by a mercury thermometer, a salinometer (Thermo Electron Corporation), and a pH meter (Model 59,003–20 USA), respectively.

### Heavy metals assessment

The evaluation of heavy metal residues (Cu, Ni, Zn, Mn, and Fe) in fish tissue and water samples was performed as follows:

### Heavy metals assessment in water specimens

A liter from each representative water specimen was delicately vaporized until complete dryness, then solubilized in 5 ml of concentrated nitric acid. Followed by incorporating an average of seven drops of hydrogen peroxide to promote wholesome digestion. Then the dry residue was dissolved in one ml of nitric acid, and the overall metals content was determined as stated by Kopp, John F and Kroner^34^.

### Heavy metals assessment in fish specimens

A half gram of ache from each fish specimen was utilized; it was heated in a muffle furnace for five hours at 450 °C. Hydrochloric acid 20% was used for the extraction process^35^. The metal’s contents were quantified by atomic absorption spectrometry (GBC Avanta E, Victoria, Australia; Ser. No. A5616).

### cDNA synthesis and quantitative real-time PCR

#### RNA extraction and cDNA synthesis

RNA was extracted from the brains, gills, kidneys, and livers of fish specimens by ABTizol (Applied Biotechnology Co. ltd, Egypt), following the manufacturer’s instructions. Where tissue samples were thoroughly homogenized in ABTizol reagent for complete cell lysis, separated by chloroform, and precipitated by isopropanol alcohol. To assess the purity and concentration of the isolated RNA, measurements were conducted by NanoDrop at 260/280 nm (BioDrop, UK). Isolated RNA from fish samples of each water body was employed in cDNA synthesis by ABT 2X RT kit (Applied Biotechnology co.ltd, Egypt), following the specified manufacturer’s instructions by adding RNA (total concentration 1ug) to 10 μl ABT2X RT Mix and completing reaction up to 20 μl with Rnase free water. The complementary DNA was preserved at − 20 °C until further analysis. The cDNA was validated by polymerase chain reaction (PCR) by using the reference gene (βeta-actin); afterwards, the amplicon was verified on 2% agarose gel electrophoreses and visualized with the gel documentation system (Biometra, Analytic Jena Company, Germany).

#### B-quantitative real-time PCR

The qRT-PCR reaction was performed to analyze the RNA transcript of growth and inflammatory-related genes (growth hormone, *gh,* and tumor necrosis factor alpha, *TNFα*) in brains; growth and oxidative stress-related genes besides heavy-metal marker gene (insulin growth factor I *IGF-I*, glutathione-S-transferase *GST,* cellular apoptosis susceptibility *cas*, and metallothionein *MT*) in liver; stress and immune-associated genes (heat shock protein 70 *HSP70*, heat shock protein 27 *HSP27*, and immunoglobulin M heavy chain *IgM*) in gills; inflammatory and immune—related genes ( interleukin 1 beta *IL1β*, interleukin8 *IL8*, CC-motif chemokine *CC*, CXC2-motif chemokine *CXC2*, and immunoglobulin M heavy chain *IgM*) in kidney. The utilized primers and sequences are listed in Table 1.

**Table 1:** Sequences of forward and reverse primers used in real time PCR.

Gene	Forward primer (5^\^–3^\^)	Reverse primer (5^\^–3^\^)	Reference
gh	TAATGGGAGAGGGAAGATGG	CTCTGCGATGTAATTCAGGA	^73^
*GST*	TAATGGGAGAGGGAAGATGG	CTCTGCGATGTAATTCAGGA	^73^
*HSP27*	CTGAGGAGCTGGTGGTGAAG	GATCAAAGGAGCCTCCACGG	^74^
*HSP70*	CTCCACCCGAATCCCCAAAA	TCGATACCCAGGGACAGAGG	^74^
*IL-1β*	AGAGCAGCAATTCAGAGC	GTGCTGATGTACCAGT	^75^
*IL-8*	GCACTGCCGCTGCATTAAG	GCAGTGGGAGTTGGGAAGAA	^76^
*CC*	ACAGAGCCGATCTTGGGTTACTTG	TGAAGGAGAGGCGGTGGATGTTAT	^77^
*C-X-C *_*2*_	CTATCCATGGAGCCTCAGGT	CTTCTTGAGCGTGGCAATAA	^78^
*IgM*	AGGAGACAGGACTGGAATGCACAA	GGAGGCAGTATAGGTATCATCCTC	^79^
*cas*	CAGTCTGTGAAAGGCCACACTATAAG	TCATTGGCTTGTGTTATTCCATGCTTCTG	^78^
*TNFα*	GGTTAGTTGAGAAGAAATCACCTGCA	GTCGTCGCTATTCCCGCAGATCA	^60^
*MT*	TTTTGTTTTCAAGGTGGAACC	AGAGGTTGGTGAACTTTGTGG	^60^
*IGFI*	TTGTCTGTGGAGAG CGAGGCT	CAGCTTTGGAAGCA GCACTCGT	^75^
*βeta-actin*	TGGCAATGAGAGGTTCCG	TGCTGTTGTAGGTGGTTTCG	^80^

The reaction mixture is composed of 10 μl SYBR green with low Rox (ABT 2X qPCR Mix (Applied Biotechnology co.ltd, Egypt), 0.8 μl of reverse and forward primer, 2 μl of cDNA, and 6.4 μl of RNase-free water (total volume 20 μl). The thermal profile started with an initial heating step for 3 min at 95 °C, followed by 45 cycles, 15 s each at 95 °C, and an annealing for 1 min at 60 °C. To confirm the precision of the PCR amplicons, the dissociation curve was created at the conclusion of the final cycle. This involved collecting fluorescence data at 60 °C and measuring every seven seconds until 95 °C.

The relative expression of the genes was calculated using comparative threshold cycle method 2^–ΔΔCT^^36^ and the results reported as fold change differences relative to the control group. βeta-actin gene was used as endogenous control for normalizer.

### Statistical analysis

Variations in results were interpreted by one-way ANOVA for heavy metal residues and two-way ANOVA for gene expression variance. GraphPad Prism software version 7.00 (Graphprism Software, La Jolla, California, USA) was used for statistical analysis. Results were expressed as mean ± SE; significance was located at *P* ≤ 0.05.

### Ethical approval

All applicable international and institutional regulations for the ethical handling and usage of animals were followed. The research protocol for the study was thoroughly examined by the National Institute of Oceanography and Fisheries (NIOF) committee for ethical care and use of animals/aquatic animals (NIOF-IACUC), and the acceptance code (NIOF-AQ6-F-23-R-045) was provided.

### ARRIVE guidelines

The authors affirm that the study was conducted in compliance with the ARRIVE guidelines.

## Results

### Water physicochemical parameters

A significantly higher temperature was observed at Lake Nasser compared to the other two aquatic environments. The recorded temperatures were 32.27, 31.43, and 30.97 for Lake Nasser, El-Qanater, and Brullus, respectively (Table 2). On the other side, salinity (ppt) recorded the highest values in Lake Brullus, followed by El-Qanater and Lake Nasser. No significant differences were observed in the pH values of the three water bodies. However, Lake Brullus tended to be alkaline (pH = 8.1), compared to the other two locations (Table 2).

**Table 2:** Physicochemical parameters of water at the three water bodies (Lake Brullus, EL-Qanater, and Lake Nasser).

	Lake Brullus	El-Qanater	Lake Nasser
Temperature	30.97 ± .4^b^	31.43 ± 0.35^b^	32.27 ± .35^a^
Salinity	5.9 ± .06^a^	0.58 ± .04^b^	0.38 ± .015^c^
PH	8.1 ± 0.4	7.66 ± 0.4	7.8 ± 0.03

Physicochemical parameters of water samples include Temperature “c^o^”, Salinity “ppt”, and Hydrogen ion concentration (pH) values.

### Analysis of heavy metal residues in water and fish tissues

Significant variations in the levels of heavy metals in water (Table 3), and fish tissues (Table 4) were observed. In addition, among the heavy metals that were examined in the three water locations, iron (Fe) had the highest residual concentration in fish tissues and water samples. In water, Lake Brullus showed the highest concentrations of Ni, Mn, Cu, and Fe, followed by EL-Qanater (River Nile) and then Lake Nasser. However, EL-Qanater showed the highest concentration of Zn compared to the other two water bodies. Remarkably, all the examined heavy metal concentrations from the three locations exceeded the maximum residual limits allowed by the World Health Organization (WHO) and the Food and Agriculture Organization (FAO). A similar pattern of heavy metal concentrations was also observed in fish tissues, where fish samples from Lake Brullus had the highest heavy metal concentrations, followed by EL-Qanater, then Lake Nasser (Table 4). Although the concentration of heavy metals in fish tissues was higher than that in water, these concentrations did not surpass the permissible limits recommended by the WHO and FAO for edible fish except for Mn.

**Table 3:** Heavy metals concentrations in water at the three water bodies (Lake Brullus, EL-Qanater, and Lake Nasser).

	Lake Brullus	El-Qanater	Lake Nasser
Fe	273.5 ± 5.500^a^	149.7 ± 1.350^b^	55.50 ± 7.500^c^
Mn	50.30 ± 2.500^a^	25.87 ± 2.854^b^	13.80 ± 1.114^c^
Zn	10.96 ± 0.840^b^	29.37 ± 2.857^a^	6.440 ± 0.969^b^
Cu	4.260 ± 0.160^a^	3.54 ± 0.422^a^	1.420 ± 0.270^b^
Ni	5.850 ± 0.150^a^	4.00 ± 0.621^b^	3.120 ± 0.288^b^

Each value represents mean ± SE of the heavy metal concentration in water (mg/L) from three different locations. Permissible limits of the studied heavy metals in water determined by WHO and FAO (**Fe**: 0.3, **Mn:** 0.1–0.2**, Zn**: 0.5- 5; **Cu**: 0.01- 0.02, **Ni**: 0.02).

**Table 4:** Heavy metals concentrations in fish tissue at the three water bodies (Lake Brullus, EL-Qanater, and Lake Nasser).

	Lake Brullus	El-Qanater	Lake Nasser
Fe	261.0 ± 3.000^a^	154.3 ± 4.509^b^	127.3 ± 3.512^c^
Mn	111.9 ± 2.139^a^	41.59 ± 1.732^b^	41.78 ± 2.924^b^
Zn	71.62 ± 2.687^a^	66.35 ± 2.179^a^	59.60 ± 2.800^b^
Cu	6.807 ± 0.469^a^	5.603 ± 0.193^b^	2.910 ± 0.271^c^
Ni	–	–	–

Each value represents mean ± SE of the heavy metal concentration in fish tissue (mg/Kg dry weight) from three different locations. Permissible limits of the studied heavy metals in fish tissue (mg/Kg dry weight) determined by WHO and FAO (**Fe**: 500–1000, **Mn:** 2–5**, Zn**: 50–150; **Cu**: 30- 50, **Ni**: 0.1- .5).

### Gene expression quantitation

**Figure 2:**
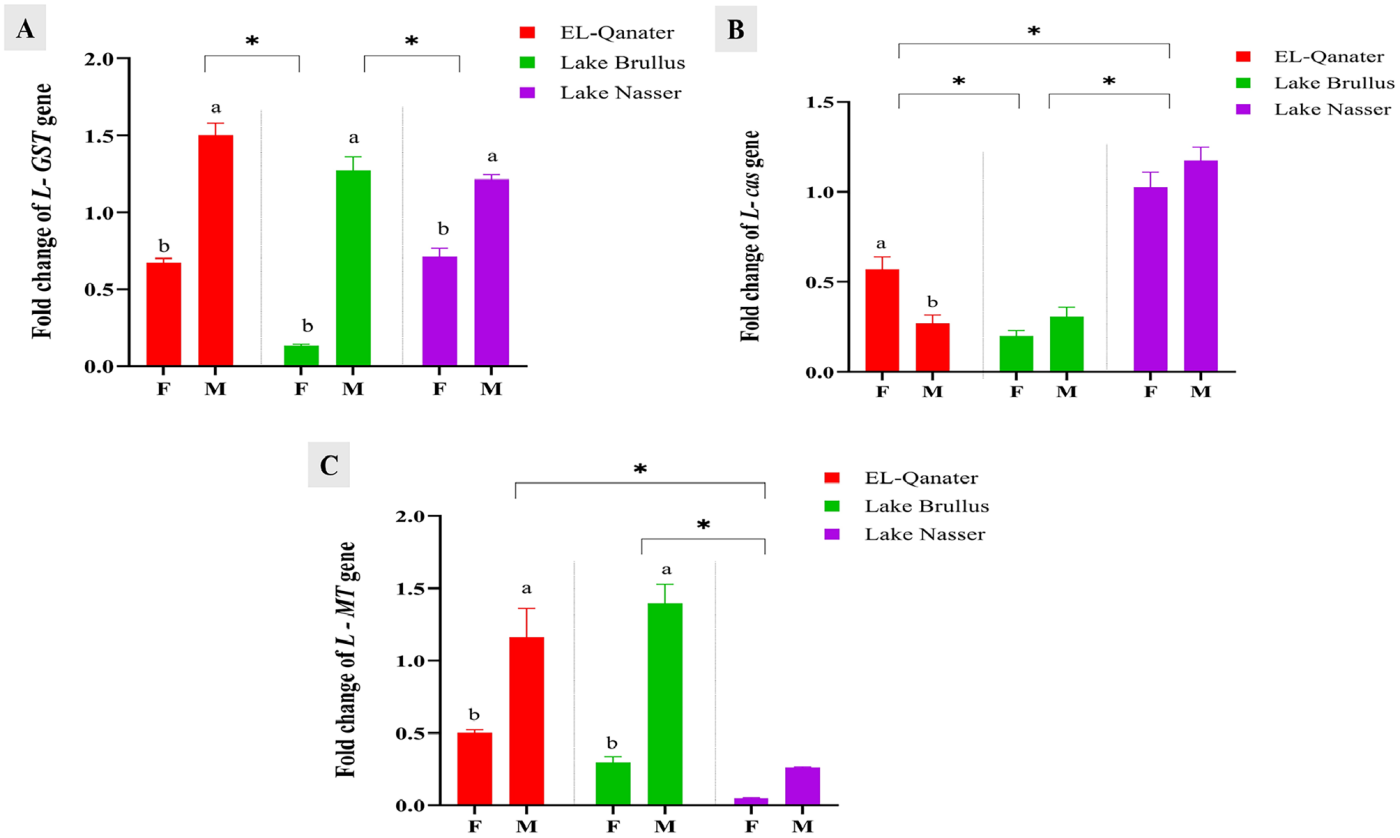
Gene expression of antioxidant genes, glutathione-s-transferase (*GST)* (**A**), cellular apoptosis susceptibility (*cas*) (**B**), and metallothionein (*MT)* (**C**) in liver of male and female Nile tilapia. Different letters indicate significant difference between male and female in same location *Indicate significant difference among the different locations. F: female, M: male.

The expression of liver antioxidant genes (*GST, cas,* and *MT*) was down-regulated in female samples at the three studied water bodies, except for the expression of *cas* at EL-Qanater (Fig. 2A–C). Additionally, the expressions of the *GST* and *MT* genes were significantly down-regulated at Lake Nasser (Fig. 2A,C). The least expression of the *MT* gene was observed in Lake Nasser in both males and females (Fig. 2C). In contrast, *cas* gene expression was significantly upregulated at Lake Nasser compared to Lake Brullus and EL-Qanater (Fig. 2B).

**Figure 3:**
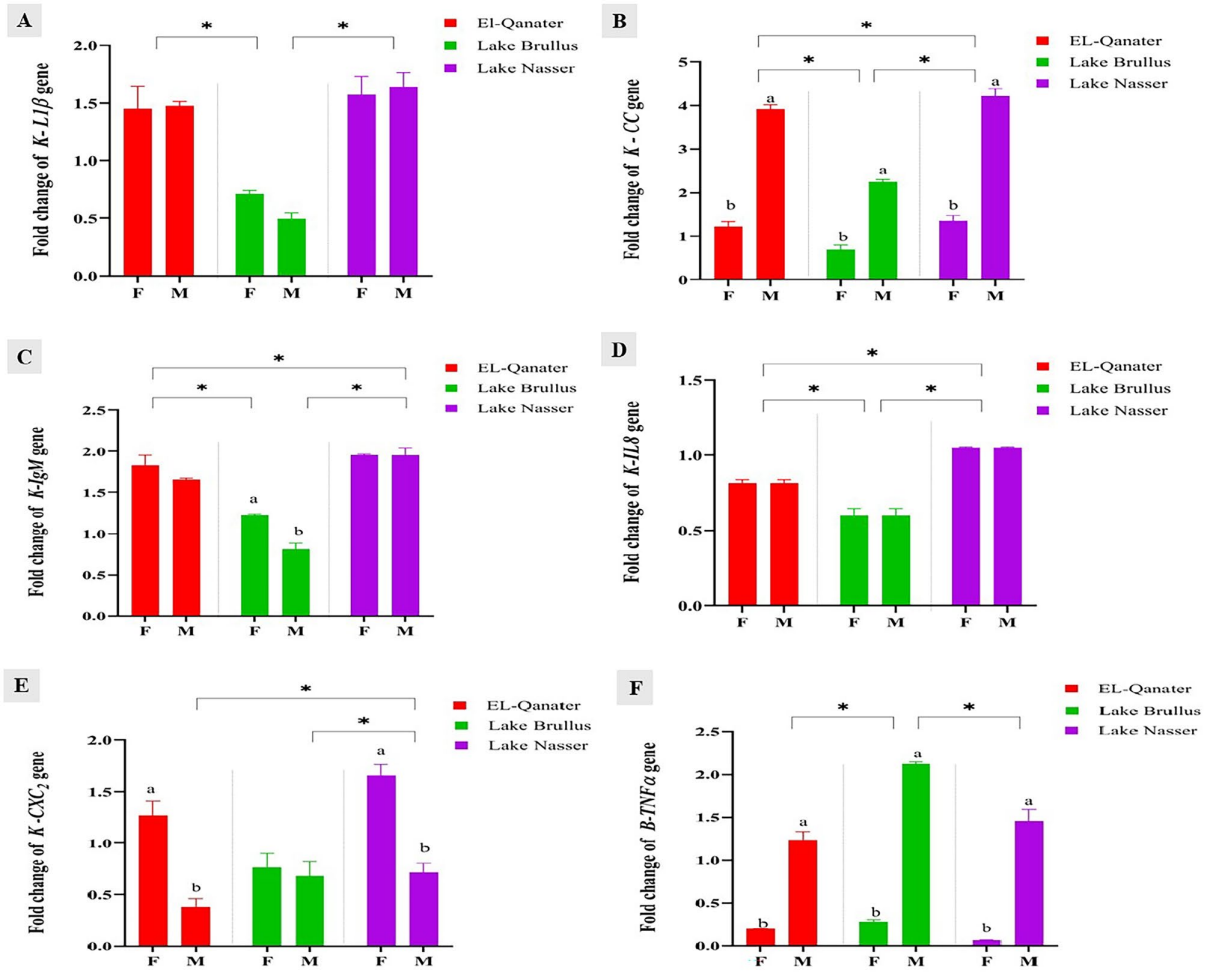
Gene expression of inflammatory and immune-related genes, interleukin 1 beta *(IL1β)* (**A**), CC-chemokine (*CC*) (**B**), Immunoglobulin M heavy chain (*IgM)* (**C**), interleukin 8 (*IL8*) (**D**), CXC2-chemokine (*CXC*_*2*_) gene (**E**) in kidney*,* and tumor necrosis factor alpha (*TNFα*) (F) in brain of male and female Nile tilapia. Different letters indicate significant difference between male and female in same location *Indicate significant difference among the different locations. F: female, M: male.

Regarding the expression of genes linked to inflammation and immunity in nephrotic and brain tissues, the gene expression of *IL1β*, *CC-chemokine*, *IgM*, *IL8,* and *CXC2-chemokine* in kidneys had a similar expression pattern at the three studied water bodies, where its expression was significantly upregulated in EL-Qanater and Lake Nasser and down-regulated at Lake Brullus (Fig. 3A–E). Furthermore, female samples showed significantly higher gene expression of *CXC2 chemokine* at El-Qanater, Lake Nasser, and of *IgM* at Lake Brullus (Fig. 3C,E). While male samples from the three studied locations showed significantly higher expression of *CC chemokine* in the kidney and *TNFα* in the brain (Fig. 3B,F). Moreover, *TNFα* was significantly upregulated at Lake Brullus compared to Lake Nasser and EL-Qanater (Fig. 3F).

**Figure 4:**
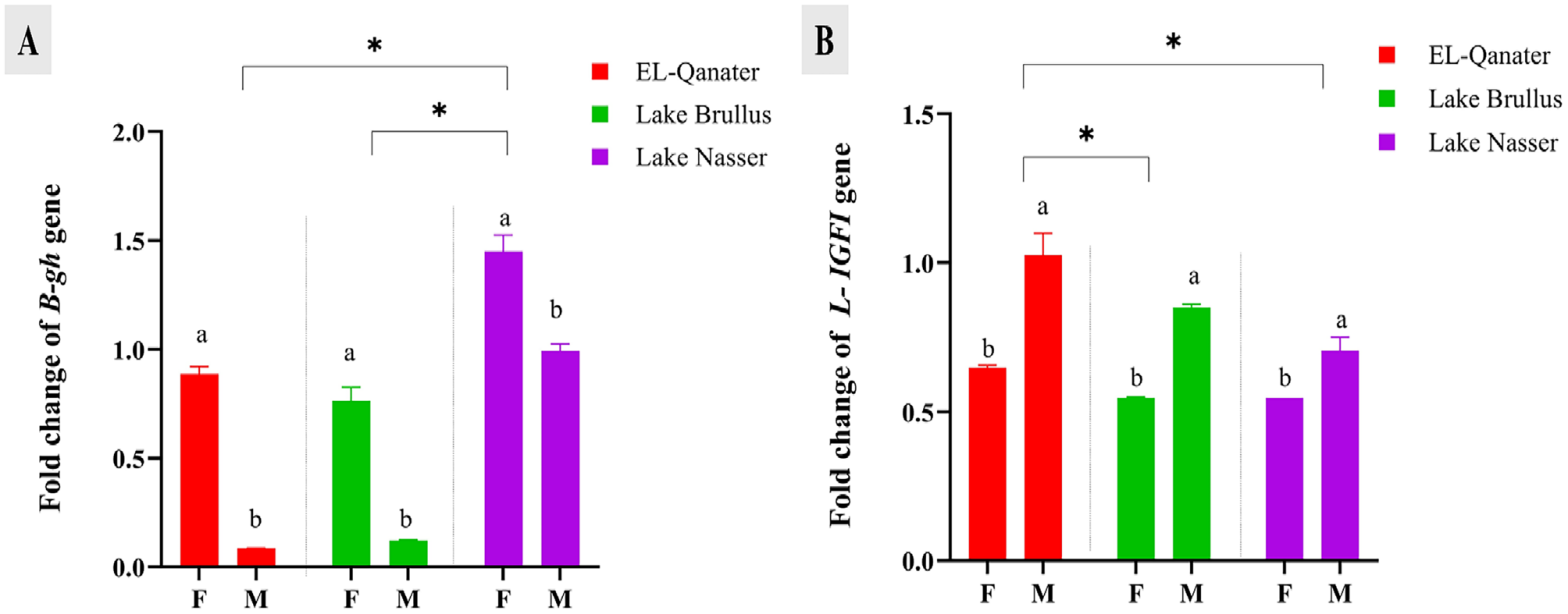
Gene expression of growth-related genes, growth hormone (*gh*) in brain (**A**) and Insulin growth factor I (*IGFI*) in liver (**B**) of male and female Nile tilapia. Different letters indicate significant difference between male and female in same location *Indicate significant difference among the different locations. F: female, M: male.

Concerning the expression of growth-related genes, the growth hormone gene, *gh,* in the brain was significantly upregulated in female samples from all locations. Moreover, Lake Nasser samples showed significantly higher expression of *gh* gene compared to samples from the other two locations (Fig. 4A). In contrast, the gene expression of the *IGFI* in the liver was significantly increased in male samples rather than females, and samples from El-Qanater showed the maximum RNA transcript of the *IGFI* gene among all locations (Fig. 4B).

**Figure 5:**
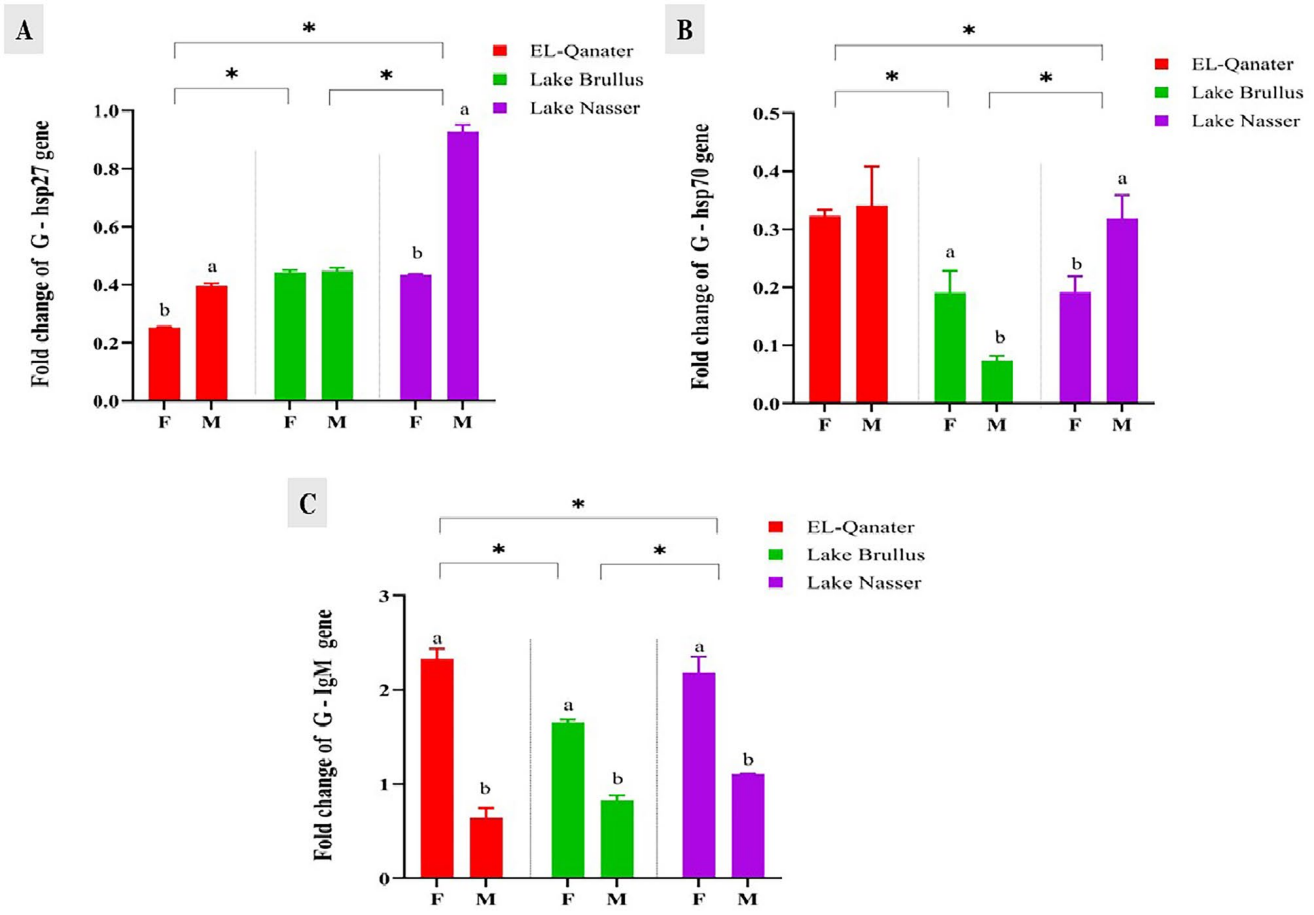
Gene expression of stress-related genes, heat shock protein 27 *(hsp27)* (**A**), heat shock protein 70 *(hsp70)* (**B**), and Immunoglobulin M heavy chain *(IgM)* (**C**) in gills of male and female Nile tilapia. Different letters indicate significant difference between male and female in same location *Indicate significant difference among the different locations. F: female, M: male.

The expression of stress and immune related genes in gills; *HSP70* and *IgM* were significantly down-regulated at Lake Brullus compared to Lake Nasser and EL-Qanater. While *HSP27* was significantly suppressed at EL-Qanater compared to the other locations, the RNA transcripts of *HSP27* and *HSP70* were significantly upregulated in males rather than females in all locations except for *HSP70* at Lake Brullus. On the other side, the expression of the *IgM* gene in gills was significantly upregulated in females at the three water bodies compared to males (Fig. 5A–C).

## Discussion

Heavy metals represent a significant menace to aquatic ecosystems owing to their deleterious effects on water quality^12^. In the current investigation, the physicochemical properties were examined in three different locations inhabited by the euryhaline and highly resistant Nile tilapia fish^3,24^. The water analysis of Lake Nasser showed the minimal salinity concentration, the highest temperature (32.27 °C), and a slightly alkaline pH (pH = 7.8). These results agree with those documented by Toufeek and Korium^37^, who found that the substantial increase in freshwater driven from the Blue Nile in the Ethiopian highlands facilitates salt distribution and lowers the lake's overall salinity^38,39^. Furthermore, Caissie^40^ and Salem^41^ reported the effects of surface heating caused by geographic location and the lesser vertical mixing of the water due to high dam construction on the elevation of the ambient temperature of the lake. It is noteworthy that the slightly alkaline pH of the lake can be due to the rise of phytoplankton photosynthetic assimilation and zooplankton growth^41^. The current study's heavy metal analysis showed that fish tissues had far greater levels of heavy metal residues than did water samples. Many studies also reported this observation; they attributed that the heavy metals enter and accumulate in the food chain directly through food and water and indirectly through the membranes of gills and muscles^15,42^. Furthermore, Fe was the most prevalent heavy metal in both fish tissues and water samples which aligns with the data published by Gjerde et al.^43^ and Javed et al.^44^, who attributed the high Fe concentrations in fish and water to the presence of many entry pathways for iron, such as ingestion, opercular pumping process, and cutaneous absorption, coupled with the absence of a specific route for full elimination. Lake Nasser displayed the lowest heavy metal concentration when compared to the other two locations. This observation is supported by Rizk et al.^45^, who reported the low heavy metal concentrations and the ideal water quality of Lake Nasser, which may refer to the crucial role of sediment in the effective collection of dissolved metals and the limited heavy metal accumulation caused only by anthropogenic biological waste^37^. On the contrary, Lake Brullus recorded the highest concentrations of heavy metals, surpassing the permissible limits in water determined by FAO^46^ and WHO^47^; salinity (5.9 ppt ± 0.06) and pH (8.1 ± 0.4). Moreover, it recorded the lowest water temperature (30.97 °C) among all locations. Similar values of pH, salinity, and low temperatures were reported by Mohamed et al.^26^ and Okbah and Hussein^48^ in Lake Brullus, and these values were referred to the continuous inflow of substantial volumes of drainage water. This steady influx not only decreases the temperature of the lake but also promotes the development of aquatic plants that eventually contribute to the depletion of dissolved oxygen levels and enhances the alkaline pH of the water^49,50^. On the other side, the recorded water temperature in the third location (El-Qanater El-Khayria) was higher than that of Lake Brullus (31.43 °C), although it is considered open water^51^. However, this higher temperature may be associated with the effect of hot water discharged from electric power stations in this region^52^.

Fish usage as a bioindicator of pollution is an efficient tool for ecosystem evaluation^11,12^. In the present research, fish samples from Lake Brullus demonstrated the highest heavy metal concentrations (Fe, Mn, Cu, and Ni) compared to the other two locations and exceeded the acceptable limits set by FAO^46^ and WHO^47,53^. This may be related to the elevated pH value of the lake, which can change heavy metal speciation and activate the development of more soluble and bioavailable compounds^54^. Additionally, the elevated salt concentration (sodium chloride (NaCl), Magnesium sulfate (MgSO₄), Calcium sulfate (CaSO₄), Calcium chloride (CaCl₂), and Sodium bicarbonate (NaHCO₃)) in the lake and the activation of the water compensation mechanism in fish may be associated with increased heavy metal exposure^55,56^. In contrast, fish collected from Lake Nasser had the lowest heavy metal concentration, which is correlated with the diminished concentration of heavy metals in the water compared to the other two locations. Additionally, Darwish^57^ demonstrated that Nasser Lake has low heavy metals level at the water and the sediment. Consequently, the biota toxicity in Lake Nasser rarely occurred.

It is well established that changes in gene expression, specifically in fish, play a pivotal role as bioindicators of heavy metal pollution^11,58–61^. In this context, the observed alteration in gene expression of genes correlated with heavy metal detoxification, immune response, and growth performance in Nile tilapia during the current research could be used as bioindicators for the aquatic ecosystem health evaluation.

Glutathione-s-transferase, *GST,* is a principal actor in the protection mechanism against oxidative stress caused by heavy metal exposure^62^. The expression level of the *GST* gene was upregulated in tilapia males, with its highest RNA transcript in EL Qanater. On the other hand, the *GST* gene was significantly downregulated in females, with its least expression in Lake Brullus in response to prolonged heavy metals exposure. These findings came in agreement with Branka Bašicaa et al.^63^, who stated that the GST enzyme showed a distinct expression pattern in response to gender and stress intensity, and it significantly increased in males compared to females under acute rather than chronic stress. Similarly, Acar et al.^64^ found a significant upregulation in Nile tilapia, *Oreochromis niloticus*, *GST* gene expression caused by acute oxidative stress. On the contrary, Abdel-Gawad et al.^65^ reported that chronic heavy metals exposure, including Zn and Cu, caused a great reduction in *GST* transcription and an increase in DNA damage in *Oreochromis niloticus*.

Cellular apoptosis susceptibility, *cas,* plays a crucial role in deciding whether a stressed cell will proliferate (*cas* induction) or encounter apoptosis (*cas* reduction)^66,67^. In Lake Nasser, in the current study, elevated transcription levels of hepatic *cas* mRNA were recorded, which was associated with the optimal water quality and the diminished heavy metal concentrations. In contrast, its expression was reduced in both male and female individuals from El Qanater and Lake Brullus, which seems to be corelated with the levels of pollutants in each water body. Likewise, Salem et al.^68^ observed a significant downregulation of the hepatic mRNA transcript of the *cas* gene in *Oreochromis niloticus* inhabiting highly polluted factory drainage with heavy metals (Fe, Cd, Mn, Pb, and Co). Similarly, Ghazy et al.^69^ found a significant downregulation of *cas* gene expression in *Oreochromis niloticus* obtained from Lake Brullus in response to heavy metal pollution*.*

Metallothionine, *MT,* is considered an ideal biomarker for heavy metal exposure; it plays a key role in metal ion transferring and detoxification processes^12,70^. In the current study, *MT* gene expression was significantly upregulated in males from Lake Brullus, which is correlated with the steep increase in heavy metal concentrations. Meanwhile, a substantial downregulation in Lake Nasser males and females was observed. This result may be assigned to the existence of a greater Zn level in the males than females, which is the strongest stimulator of *MT* synthesis and arises from the inductive impact of the ZIP9 androgen-activated membrane receptor^70^. Similarly, El-Sayed et al.^58^ noticed a significant upregulation of the hepatic *MT* gene in *Oreochromis niloticus* upon heavy metal exposure.

The brain perceives stress as an early warning sign, which stimulates innate immunity through the activation of proinflammatory cytokines and chemokines expression. Whereas chronic stressors deplete the body and suppress immune responses, increasing the risk of infection^71^. Accordingly, in the current investigation, the gene expression of pro-inflammatory and immune-related genes (*IL1β, CC-chemokine, IL8, IgM,* and *CXC*_*2*_) in the kidney in addition to *IgM* in gills were significantly upregulated in Lake Nasser and EL-Qanater and downregulated in Lake Brullus, reflecting the variable levels of oxidative stress at the studied locations, starting from Lake Nasser, followed by El-Qanater EL-khayria, and Lake Brullus. These results came in accordance with Zahran et al.^59^, who found a significant rise in *IL1β* mRNA gene expression in Nile tilapia, *Oreochromis niloticus,* after acute arsenic exposure. Moreover, Wang et al.^72^ discovered a significant decrease in *IL1β* mRNA transcription in *Oreochromis niloticus* exposed to chronic oxidative stress generated by Cu and Zn. Additionally, Abo-Al-Ela et al.^73^ discovered a considerable increase in *IL1β, IL8, CC chemokine, CXC2 chemokine,* and *IgM* in *Oreochromis niloticus* fry after exposure to oxidative stress caused by 17 alpha-methyltestosterone for 14 days. Furthermore, the *IgM* and *CXC2* gene expression was significantly upregulated in females and suppressed in males. These findings can be attributed to the sex related responses of innate immunity, where females are equipped with very sensitive immune responses due to the presence of estrogen receptors on immune cells, while males display a weaker immune response due to androgen hypothalamus–pituitary–adrenal axis inhibition^22^.

Tumer necrosis factor alpha, *TNFα,* is a hormone-suppressed pro-inflammatory cytokine. It performs a crucial role in the immune response, attracting immune cells and controlling apoptosis in fish in response to environmental stresses^74–76^. In the present study, the expression of *TNFα* gene in the brain was induced in males, with the maximum expression in Lake Brullus, favoring cell apoptosis in response to intense oxidative stress generated by elevated heavy metal concentrations. In contrast, *TNFα* gene expression was suppressed in females with the minimum expression in Lake Nasser. These findings may refer to the hormonal control of *TNFα*, where estrogen, the primary female sex hormone in teleost, possesses a suppressive effect on *TNFα* expression^75^. Yin et al.^76^ recorded a significant upregulation of *TNF-α* and *IL1β* genes in a progressive manner after exposure to chromium (Cr), lead (Pb), copper (Cu), and cadmium (Cd) in adult zebrafish, *Danio rerio*. Also, Cobbina et al.^77^ investigated the effect of individual and combined heavy metals on the larval stage of zebrafish, *Danio rerio*. *TNFα* expression was strongly decreased by arsenic and a combination of lead and arsenic when compared to control.

Growth performance is physiologically linked to stress, enabling fish to modify their metabolism to meet energy demands and maintain homeostasis^78^. In the current study, growth hormone, *gh,* gene expression in brain tissue was significantly repressed in El-Qanater and Lake Brullus in an energy-shifting mechanism to correct oxidative stress damage. Also, hepatic *IGFI* gene expression was dramatically reduced, with the least amount of mRNA transcripts detected in Lake Brullus. Furthermore, males showed a significant downregulation in the expression of the *gh* gene, while *IGFI* was significantly upregulated in comparison to females. The reduction in *gh* gene expression might be attributed to the significant rise in *TNFα* levels in males, as *TNFα* is recognized to promote hepatic resistance to growth hormone in fish^79^. On the other side, the induction of *IGFI* may be caused by the role of *IGFI* as a local regulator of fish spermatogenesis^80^. Likewise, Hu et al.^60^ reported that the long-term exposure of *Oreochromis niloticus* to Cd dramatically lowered body length and weight and down-regulated hepatic mRNA levels of *IGFI*, *IGFII,* and *GHRs*. Furthermore, Zebral et al.^81^ reported that long-term copper (Cu2 +) exposure in the fish Poecilia vivipara*, Teleostei Poeciliidae,* resulted in muscle growth hormone insensitivity and decreased muscular *IGFI* and *IGFII* mRNA expression. Moreover, Taslima et al.^61^ reported a reduction in the larval and embryonic survival rate, delayed hatching, dwarfism, and morphological abnormalities of African catfish, *Clarias gariepinus*, larvae on exposure to heavy metal pollution.

Heat shock proteins *HSPs* are synthesized in response to a wide range of acute stressors and metabolic disturbances to achieve cellular homeostasis by preventing the development of nonspecific protein aggregates^22^. In the present study, the gene expression of *HSP27* and *HSP70* in gills were significantly downregulated in Lake Brullus and EL-Qanater compared to Lake Nasser, which may be attributed to the chronic heavy metals’ exposure at these locations. Furthermore, males showed a significant upregulation in the expression of the *HSP27* gene compared to females. This rise in *HSP27 expression* may be caused by the physiological difference between males and females, as *HSPs* are expressed more highly in the testicles than in the ovaries^82^. Similarly, the expression of *HSP90* and *HSP70* was significantly suppressed in gilthead sea bream, *Sparus aurata,* samples exposed to sediments with high metal concentrations^83^. Furthermore, Luo et al.^84^ discovered a significant suppression of *HSPs* in the gills of oysters, *Crassostrea hongkongensis*, inhabiting heavy metal-contaminated environments.

## Conclusion

In conclusion, the present study clarifies that heavy metals’ bioavailability is influenced by their chemical properties and ambient environmental conditions. The concentration of heavy metal residues in the three studied locations greatly affects the water quality of the aquatic ecosystem, which is reflected in fish health and consequently may affect human health. Lake Brullus emerged as the most polluted area, exhibiting elevated heavy metal concentrations in water and fish tissue, necessitating prompt attention and corrective action to preserve both aquatic life and human health in the surrounding areas. Lake Nasser showed the least level of pollution, which was reflected in the normal gene expression levels and the increased growth hormone gene expression in its fish samples. A potential gender-specific gene expression response of antioxidant and inflammatory-related genes to pollution was recorded. This points out the significance of considering gender-specific responses in future environmental evaluations. As the biological sex can influence how aquatic react to climate change. Enhancing climate adaptation and mitigation efforts requires this new knowledge.

## Data Availability

The datasets utilized and/or analysis performed during the current investigation are accessible from the corresponding author on reasonable request.
